# Reasoning over Permissions Regions in Concurrent Separation Logic

**DOI:** 10.1007/978-3-030-53291-8_13

**Published:** 2020-06-16

**Authors:** James Brotherston, Diana Costa, Aquinas Hobor, John Wickerson

**Affiliations:** 8 Microsoft Research Lab, Redmond, WA USA; 9 University of Southern California, Los Angeles, CA USA; 10 University College London, London, UK; 11 National University of Singapore, Singapore, Singapore; 12 Imperial College London, London, UK

**Keywords:** Separation logic, Permissions, Concurrency, Verification

## Abstract

We propose an extension of separation logic with fractional permissions, aimed at reasoning about concurrent programs that share arbitrary *regions* or data structures in memory. In existing formalisms, such reasoning typically either fails or is subject to stringent side conditions on formulas (notably *precision*) that significantly impair automation. We suggest two formal syntactic additions that collectively remove the need for such side conditions: first, the use of both “weak” and “strong” forms of separating conjunction, and second, the use of nominal labels from hybrid logic. We contend that our suggested alterations bring formal reasoning with fractional permissions in separation logic considerably closer to common pen-and-paper intuition, while imposing only a modest bureaucratic overhead.

## Introduction

*Concurrent separation logic* ($\mathsf {CSL}$) is a version of separation logic designed to enable compositional reasoning about concurrent programs that manipulate memory possibly shared between threads [6, 26]. Like standard separation logic [28], $\mathsf {CSL}$ is based on *Hoare triples* , where *C* is a program and *A* and *B* are formulas (called the *precondition* and *postcondition* of the code respectively). The heart of the formalism is the following *concurrency rule*: 
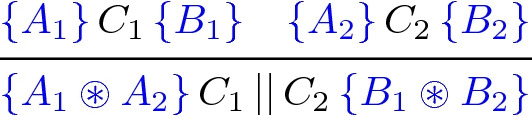
where $\circledast$ is a so-called *separating conjunction*. This rule says that if two threads $C_1$ and $C_2$ are run on spatially separated resources $A_1 \circledast A_2$ then the result will be the spatially separated result, $B_1 \circledast B_2$, of running the two threads individually.

However, since many or perhaps even most interesting concurrent programs do share some resources, $\circledast$ typically does not denote strict disjoint separation of memories, as it does in standard separation logic (where it is usually written as $*$). Instead, it usually denotes a weaker sort of “separation” designed to ensure that the two threads at least cannot interfere with each others’ data. This gives rise to the idea of *fractional permissions*, which allow us to divide writeable memory into multiple read-only copies by adding a permission value to each location in heap memory. In the usual model, due to Boyland [5], permissions are rational numbers in the half-open interval (0, 1], with 1 denoting the write permission, and values in (0, 1) denoting read-only permissions. We write the formula $A^\pi$, where $\pi$ is a permission, to denote a “$\pi$ share” of the formula *A*. For example, $(x \mapsto a)^{0.5}$ (typically written as  for convenience) denotes a “half share” of a single heap cell, with address *x* and value *a*. The separating conjunction $A \circledast B$ then denotes heaps realising *A* and *B* that are “compatible”, rather than disjoint: where the heaps overlap, they must agree on the data value, and one adds the permissions at the overlapping locations [4]. E.g., at the logical level, we have the entailment:1Happily, the concurrency rule of $\mathsf {CSL}$ is still sound in this setting (see e.g. [29]).

However, the use of this weaker notion of separation $\circledast$ causes complications for formal reasoning in separation logic, especially if one wishes to reason over arbitrary regions of memory rather than individual pointers. There are two particular difficulties, as identified by Le and Hobor [24]. The first is that, since $\circledast$ denotes possibly-overlapping memories, one loses the main useful feature of separation logic: its nonambiguity about separation, which means that desirable entailments such as $A^{0.5} \circledast B^{0.5} \models (A \circledast B)^{0.5}$ turn out to be false. E.g.:Here, the two “half-pointers” on the LHS might be aliased ($x=y$ and $a=b$), meaning they are two halves of the same pointer, whereas on the RHS they must be non-aliased (because we cannot combine two “whole” pointers). This ambiguity becomes quite annoying when one adds arbitrary predicate symbols to the logic, e.g. to support inductively defined data structures.

The second difficulty is that although recombining single pointers is straightforward, as indicated by Eq. (1), recombining the shares of arbitrary formulae is challenging. E.g., $A^{0.5} \circledast A^{0.5} \not \models A$, as shown by the counterexample

$$\begin{aligned} (x \mapsto 1 \vee y \mapsto 2)^{0.5} \circledast (x \mapsto 1 \vee y \mapsto 2)^{0.5} \not \models x \mapsto 1 \vee y \mapsto 2. \end{aligned}$$

The LHS can be satisfied by a heap with a 0.5-share of *x* and a 0.5-share of *y*, whereas the RHS requires a full (1) share of either *x* or *y*.

Le et al. [24] address these problems by a combination of the use of *tree shares* (essentially Boolean binary trees) rather than rational numbers as permissions, and semantic restrictions on when the above sorts of permissions reasoning can be applied. For example, recombining permissions ($A^{0.5} \circledast A^{0.5} \models A$) is permitted only when the formula is *precise* in the usual separation logic sense (cf. [28]). The chief drawback with this approach is the need to repeatedly check these side conditions on formulas when reasoning, as well as that said reasoning cannot be performed on imprecise formulas.

Instead, we propose to resolve these difficulties by a different, two-pronged extension to the syntax of the logic. First, we propose that the usual “strong” separating conjunction $*$, which enforces the strict disjointness of memory, *should be retained* in the formalism in addition to the weaker $\circledast$. The stronger $*$ supports entailments such as $A^{0.5} * B^{0.5} \models (A * B)^{0.5}$, which does not hold when $\circledast$ is used instead. Second, we introduce *nominal labels* from hybrid logic (cf. [3, 10]) to remember that two copies of a formula have the same origin. We write a nominal $\alpha$ to denote a unique heap, in which case entailments such as $(\alpha \wedge A)^{0.5} \circledast (\alpha \wedge A)^{0.5} \models \alpha \wedge A$ become valid. We remark that labels have been adopted for similar “tracking” purposes in several other separation logic proof systems [10, 21, 23, 25].

The remainder of this paper aims to demonstrate that our proposed extensions are (i) weakly *necessary*, in that expected reasoning patterns fail under the usual formalism, (ii) *correct*, in that they recover the desired logical principles, and (iii) *sufficient* to verify typical concurrent programming patterns that use sharing. Section 2 gives some simple examples that motivate our extensions. Section 3 then formally introduces the syntax and semantics of our extended formalism. In Sect. 4 we show that our logic obeys the logical principles that enable us to reason smoothly with fractional permissions over arbitrary formulas, and in Sect. 5 we give some longer worked examples. Finally, in Sect. 6 we conclude and discuss directions for future work.

## Motivating Examples

In this section, we aim to motivate our extensions to separation logic with permissions by showing, firstly, how the failures of the logical principles described in the introduction actually arise in program verification examples and, secondly, how these failures are remedied by our proposed changes.

The overall context of our work is reasoning about concurrent programs that share some data structure or region in memory, which can be described as a formula in the assertion language. If *A* is such a formula then we write $A^\pi$ to denote a “$\pi$ share” of the formula *A*, meaning informally that all of the pointers in the heap memory satisfying *A* are owned with share $\pi$. The main question then becomes how this notion interacts with the separating conjunction $\circledast$. There are two key desirable logical equivalences:

**Figure:**
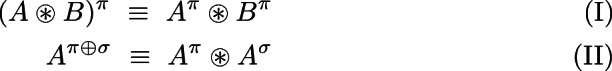

Equivalence (I) describes distributing a fractional share over a separating conjunction, whereas equivalence (II) describes combining two pieces of a previously split resource. Both equivalences are true in the $\models$ direction but, as we have seen in the Introduction, false in the  one. Generally speaking, $\circledast$ is like Humpty Dumpty: easy to break apart, but not so easy to put back together again.

The key to understanding the difficulty is the following equivalence:In other words, either *x* and *y* are not aliased, or they *are* aliased and the permissions combine (the additive operation $\oplus$ on rational shares is simply normal addition when the sum is $\le 1$ and undefined otherwise). This disjunction undermines the notational economies that have led to separation logic’s great successes in scalable verification [11]; in particular, (I) fails because the left disjunct might be true, and (II) fails because the right disjunct might be. At a high level, $\circledast$ is a bit too easy to introduce, and therefore also a bit too hard to eliminate.

### Weak vs. Strong Separation and the Distribution Principle

One of the challenges of the weak separating conjunction $\circledast$ is that it interacts poorly with inductively defined predicates. Consider porting the usual separation logic definition of a possibly-cyclic linked list segment from *x* to *y* from a sequential setting to a concurrent one by a simple substitution of $\circledast$ for $*$: 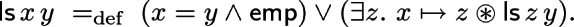
Now consider a simple recursive procedure $\texttt {foo(x,y)}$ that traverses a linked list segment from $\texttt {x}$ to $\texttt {y}$:It is easy to see that $\texttt {foo}$ leaves the list segment unchanged, and therefore satisfies the following Hoare triple: 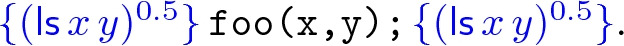
The intuitive proof of this fact would run approximately as follows: 
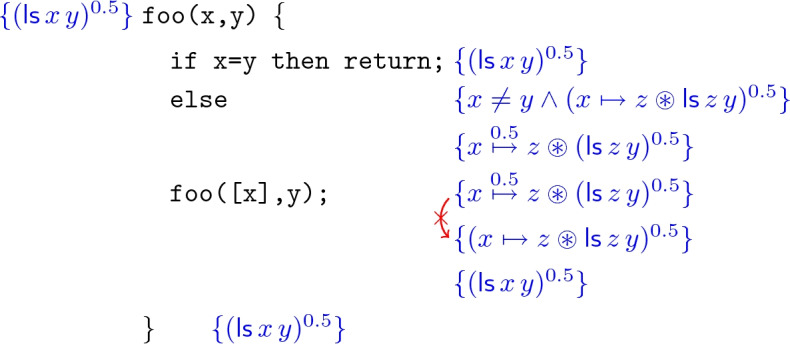
However, because of the use of $\circledast$, the highlighted inference step is not sound:2To see this, consider a heap with the following structure, viewed in two ways:

$$ x {\mathop {\mapsto }\limits ^{0.5}} z \circledast z {\mathop {\mapsto }\limits ^{0.5}} x \circledast x {\mathop {\mapsto }\limits ^{0.5}} z \;\; = \;\; x \mapsto z \circledast z {\mathop {\mapsto }\limits ^{0.5}} x $$

This heap satisfies the LHS of the entailment in (2), as it is the $\circledast$-composition of a 0.5-share of $x \mapsto z$ and a 0.5-share of , a cyclic list segment from *z* back to itself (note that here $z=y$). However, it does not satisfy the RHS, since it is not a 0.5-share of the $\circledast$-composition of $x \mapsto z$ with , which would require the pointer to be disjoint from the list segment.

The underlying reason for the failure of this example is that, in going from  to , we have lost the information that the pointer and the list segment are actually disjoint. This is reflected in the general failure of the distribution principle $A^\pi \circledast B^\pi \models (A \circledast B)^\pi$, of which the above is just one instance. Accordingly, our proposal is that the “strong” separating conjunction $*$ from standard separation logic, which forces disjointness of the heaps satisfying its conjuncts, should *also* be retained in the logic alongside $\circledast$, on the grounds that (II) *is* true for the stronger connective:

3 $$\begin{aligned} (A * B)^\pi \equiv A^\pi * B^\pi . \end{aligned}$$

If we then define our list segments using $*$ in the traditional way, namely 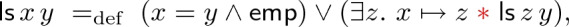
then we can observe that this second definition of $\mathsf {ls}$ is identical to the first on permission-free formulas, since $\circledast$ and $*$ coincide in that case. However, when we replay the verification proof above with the new definition of $\mathsf {ls}$, every $\circledast$ in the proof above becomes a $*$, and the proof then becomes sound. Nevertheless, we can still use $\circledast$ to describe permission-decomposition of list segments at a higher level; e.g.,  can still be decomposed as $(\mathsf {ls}\,x\,y)^{0.5} \circledast (\mathsf {ls}\,x\,y)^{0.5}$.

### Nominal Labelling and the Combination Principle

Unfortunately, even when we use the strong separating conjunction $*$ to define list segments $\mathsf {ls}$, a further difficulty still remains. Consider a simple concurrent program that runs two copies of $\texttt {foo}$ in parallel on the same list segment:

$$ \texttt {foo(x,y);} \;\;||\;\; \texttt {foo(x,y);} $$

Since $\texttt {foo}$ only reads from its input list segment, and satisfies the specification , this program satisfies the specification 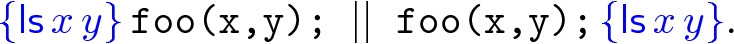
Now consider constructing a proof of this specification in $\mathsf {CSL}$. First we view the list segment  as the $\circledast$-composition of two read-only copies, with permission 0.5 each; then we use $\mathsf {CSL}$’s concurrency rule (see Sect. 1) to compose the specifications of the two threads; last we recombine the two read-only copies to obtain the original list segment. The proof diagram is as follows: 
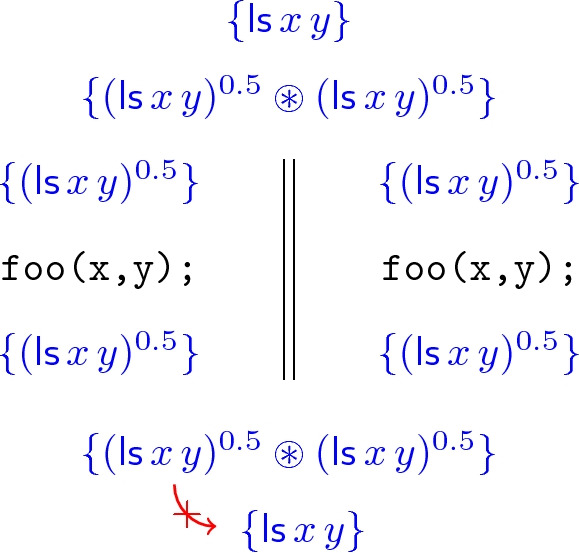
However, again, the highlighted inference step in this proof is not correct:4A countermodel is a heap with the following structure, again viewed in two ways:

$$ (x {\mathop {\mapsto }\limits ^{0.5}} y \circledast y {\mathop {\mapsto }\limits ^{0.5}} y) \circledast x {\mathop {\mapsto }\limits ^{0.5}} y \;\; = \;\; x \mapsto y \circledast y {\mathop {\mapsto }\limits ^{0.5}} y $$

According to the first view of such a heap, it satisfies the LHS of (4), as it is the $\circledast$-composition of two 0.5-shares of  (one of two cells, and one of a single cell). However, it does not satisfy , since that would require every cell in the heap to be owned with permission 1.

Like in our previous example, the reason for the failure of this example is that we have lost information. In going from  to $(\mathsf {ls}\,x\,y)^{0.5} \circledast (\mathsf {ls}\,x\,y)^{0.5}$, we have forgotten that the two formulas $(\mathsf {ls}\,x\,y)^{0.5}$ are in fact *copies of the same region*. For formulas *A* that are *precise* in that they uniquely describe part of any given heap [12], e.g. formulas $x \mapsto a$, this loss of information does not happen and we do have $A^{0.5} \circledast A^{0.5} \models A$; but for non-precise formulas such as , this principle fails.

However, we regard this primarily as a technical shortcoming of the formalism, rather than a failure of our intuition. It *ought* to be true that we can take any region of memory, split it into two read-only copies, and then later merge the two copies to re-obtain the original region. Were we conducting the above proof on pen and paper, we would very likely explain the difficulty away by adopting some kind of labelling convention, allowing us to remember that two formulas have been obtained from the same memory region by dividing permissions.

In fact, that is almost exactly our proposed remedy to the situation. We introduce *nominals*, or *labels*, from hybrid logic, where a nominal $\alpha$ is interpreted as denoting a unique heap. Any formula of the form $\alpha \wedge A$ is then precise (in the above sense), and so obeys the combination principle

5 $$\begin{aligned} (\alpha \wedge A)^{\pi } \circledast (\alpha \wedge A)^{\sigma } \models (\alpha \wedge A)^{\sigma \oplus \pi } , \end{aligned}$$

where $\oplus$ is addition on permissions. Thus we can repair the faulty $\mathsf {CSL}$ proof above by replacing every instance of the formula  by the “labelled” formula  (and adding an initial step in which we introduce the fresh label $\alpha$).

### The Jump Modality

However, this is not quite the end of the story. Readers may have noticed that replacing  by the “labelled” version  also entails establishing a slightly stronger specification for the function $\texttt {foo}$, namely: 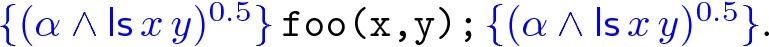
This introduces an extra difficulty in the proof (cf. Sect. 2.1); at the recursive call to $\texttt {foo([x],y)}$, the precondition now becomes , which means that we cannot apply separation logic’s *frame rule* [32] to the pointer formula without first weakening away the label-share $\alpha ^{0.5}$.

For this reason, we shall also employ hybrid logic’s “jump” modality $@_{\_}$, where the formula $@_\alpha A$ means that *A* is true of the heap denoted by the label $\alpha$. In the above, we can introduce labels $\beta$ and $\gamma$ for the list components $x \mapsto z$ and  respectively, whereby we can represent the decomposition of the list by the assertion $@_\alpha (\beta * \gamma )$. Since this is a *pure* assertion that does not depend on the heap, it can be safely maintained when applying the frame rule, and used after the function call to restore the label $\alpha$, using the easily verifiable fact that

$$ @_\alpha (\beta * \gamma ) \wedge (\beta * \gamma ) \models \alpha . $$

Similar reasoning over labelled decompositions of data structures is seemingly necessary whenever treating recursion; we return to it in more detail in Sect. 5.

## Separation Logic with Labels and Permissions ($\mathsf {SL}_{\mathsf {LP}}$)

Following the motivation given in the previous section, here we give the syntax and semantics of a separation logic, $\mathsf {SL}_{\mathsf {LP}}$, with permissions over arbitrary formulas, making use of both strong *and* weak separating conjunctions, and nominal labels (from hybrid logic [3, 10]). First, we define a suitable notion of permissions and associated operations.

### Definition 3.1

A *permissions algebra* is a tuple $\langle \mathsf{Perm}, \oplus , \otimes , 1 \rangle$, where $\mathsf{Perm}$ is a set (of “permissions”), $1 \in \mathsf{Perm}$ is called the *write permission*, and $\oplus$ and $\otimes$ are respectively partial and total binary functions on $\mathsf{Perm}$, satisfying associativity, commutativity, cancellativity and the following additional axioms:

$$\begin{array}{l@{}l} \pi _1 \oplus \pi _2 \ne \pi _2 &{} \text{(non-zero) } \\ \forall \pi .\ \pi \oplus 1 \text{ is } \text{ undefined } &{} \text{(top) } \\ \forall \pi .\ \exists \pi _1,\pi _2.\ \pi = \pi _1 \oplus \pi _2 &{} \text{(divisibility) } \\ (\pi _1\oplus \pi _2)\otimes \pi = (\pi _1 \otimes \pi )\oplus (\pi _2 \otimes \pi ) &{} \text{(left-dist) } \\ \end{array}$$

The most common example of a permissions algebra is the Boyland fractional permission model $\langle (0,1]\cap \mathbb {Q}, \oplus , \times , 1\rangle$, where permissions are rational numbers in (0, 1], $\times$ is standard multiplication, and $\oplus$ is standard addition but undefined if $p+p' > 1$. From now on, we assume a fixed but arbitrary permissions algebra.

With the permissions structure in place, we can now define the syntax of our logic. We assume disjoint, countably infinite sets $\mathsf {Var}$ of variables, $\mathsf {Pred}$ of predicate symbols (with associated arities) and $\mathsf {Label}$ of labels.

### Definition 3.2

We define *formulas* of $\mathsf {SL}_{\mathsf {LP}}$ by the grammar: 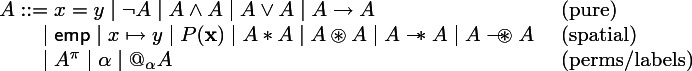
where *x*, *y* range over $\mathsf {Var}$, $\pi$ ranges over $\mathsf{Perm}$, *P* ranges over $\mathsf {Pred}$, $\alpha$ ranges over $\mathsf {Label}$ and $\mathbf {x}$ ranges over tuples of variables of length matching the arity of the predicate symbol *P*. We write  for $(x \mapsto y)^\pi$, and $x \ne y$ for $\lnot (x=y)$.

The “magic wands”  and  are the implications adjoint to $*$ and $\circledast$, as usual in separation logic. We include them for completeness, but we use  only for fairly complex examples (see Sect. 5.3) and in fact do not use  at all.

*Semantics.* We interpret formulas in a standard model of stacks and heaps-with-permissions (cf. [4]), except that our models also incorporate a valuation of nominal labels. We assume an infinite set $\mathsf{Val}$ of *values* of which an infinite subset $\mathsf{Loc}\subset \mathsf{Val}$ are considered addressable *locations*. A *stack* is as usual a map $s : \mathsf {Var}\rightarrow \mathsf{Val}$. A *heap-with-permissions*, which we call a *p-heap* for short, is a finite partial function $h : \mathsf{Loc}\rightharpoonup _{\mathrm {fin}} \mathsf{Val}\times \mathsf{Perm}$ from locations to value-permission pairs. We write $\mathrm {dom}\left( h\right)$ for the *domain* of *h*, i.e. the set of locations on which *h* is defined. Two p-heaps $h_1$ and $h_2$ are called *disjoint* if $\mathrm {dom}\left( h_1\right) \cap \mathrm {dom}\left( h_2\right) =\emptyset$, and *compatible* if, for all $\ell \in \mathrm {dom}\left( h_1\right) \cap \mathrm {dom}\left( h_2\right)$, we have $h_1(\ell ) = (v,\pi _1)$ and $h_2(v,\pi _2)$ and $\pi _1 \oplus \pi _2$ is defined. (Thus, trivially, disjoint heaps are also compatible.) We define the multiplication $\pi \cdot h$ of a p-heap *h* by permission $\pi$ by extending $\otimes$ pointwise:

$$ (\pi \cdot h)(\ell ) = (v, \pi \otimes \pi ') \;\;\Leftrightarrow \;\; h(\ell ) = (v,\pi '). $$

We also assume that each predicate symbol *P* of arity *k* is given a fixed interpretation $\llbracket P\rrbracket \in (\mathsf{Val}^k \times \mathsf {PHeaps})$, where $\mathsf {PHeaps}$ is the set of all p-heaps. Here we allow an essentially free interpretation of predicate symbols, but they could also be given by a suitable inductive definition schema, as is done in many papers on separation logic (e.g. [7, 8]). Finally, a *valuation* is a function $\rho :\mathsf {Label}\rightarrow \mathsf {PHeaps}$ assigning a single p-heap $\rho (\alpha )$ to each label $\alpha$.

### Definition 3.3

**(Strong and weak heap composition).** The *strong composition* $h_1 \circ h_2$ of two disjoint p-heaps $h_1$ and $h_2$ is defined as their union: 
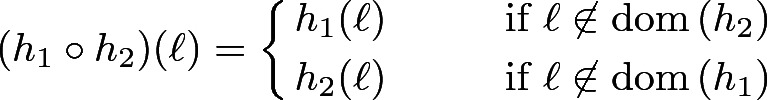
If $h_1$ and $h_2$ are not disjoint then $h_1 \circ h_2$ is undefined.

The *weak composition*  of two compatible p-heaps $h_1$ and $h_2$ is defined as their union, adding permissions at overlapping locations: 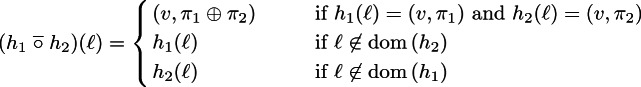
If $h_1$ and $h_2$ are not compatible then  is undefined.

### Definition 3.4

**Fig. 1.**
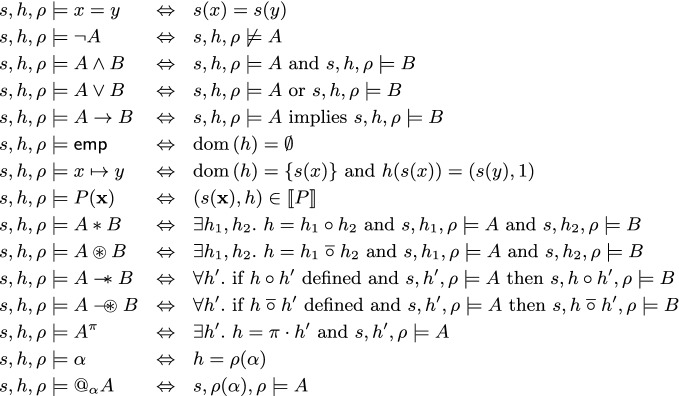
Definition of the satisfaction relation $s,h,\rho \models A$ for $\mathsf {SL}_{\mathsf {LP}}$.

The satisfaction relation $s,h,\rho \models A$, where *s* is a stack, *h* a p-heap, $\rho$ a valuation and *A* a formula, is defined by structural induction on *A* in Fig. 1. We write the *entailment* $A \models B$, where *A* and *B* are formulas, to mean that if $s,h,\rho \models A$ then $s,h,\rho \models B$. We write the *equivalence* $A \equiv B$ to mean that $A \models B$ and $B \models A$.

## Logical Principles of $\mathsf {SL}_{\mathsf {LP}}$

In this section, we establish the main logical entailments and equivalences of $\mathsf {SL}_{\mathsf {LP}}$ that capture the various interactions between the separating conjunctions $\circledast$ and $*$, permissions and labels. As well as being of interest in their own right, many of these principles will be essential in treating the practical verification examples in Sect. 5. In particular, the permission distribution principle for $*$ (cf. (3), Sect. 2) is given in Lemma 4.3, and the permission combination principle for labelled formulas (cf. (5), Sect. 2) is given in Lemma 4.4.

### Proposition 4.1

The following equivalences all hold in $\mathsf {SL}_{\mathsf {LP}}$:

$$\begin{array}{rcl@{}rcl} A \circledast B &{} \!\!\!\equiv &{}\!\!\! B \circledast A &{} A * B &{}\!\!\! \equiv &{}\!\!\! B * A \\ A \circledast (B \circledast C) &{}\!\!\! \equiv &{}\!\!\! (A \circledast B) \circledast C &{} A * (B * C) &{}\!\!\! \equiv &{}\!\!\! (A * B) * C \\ A \circledast \mathsf {emp}&{}\!\!\! \equiv &{}\!\!\! A &{} A * \mathsf {emp}&{}\!\!\! \equiv &{}\!\!\! A \\ \end{array}$$

Additionally, the following residuation laws hold: 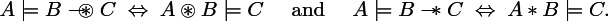
In addition, we can always weaken $*$ to $\circledast$:  $A * B \models A \circledast B$.

Next, we establish an additional connection between the two separating conjunctions $\circledast$ and $*$.

### Lemma 4.2

**(**$\circledast /*$ **distribution).** For all formulas *A*, *B*, *C* and *D*, 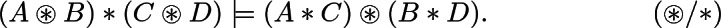


### Proof

First we show a corresponding model-theoretic property: for any p-heaps $h_1, h_2, h_3$ and $h_4$ such that  is defined,6Since  is defined by assumption, we have that  and  are disjoint and that $h_1$ and $h_2$, as well as $h_3$ and $h_4$ are compatible. In particular, $h_1$ and $h_3$ are disjoint, so $h_1\circ h_3$ is defined; the same reasoning applies to $h_2$ and $h_4$. Moreover, since $h_1$ and $h_2$ are compatible, $h_1\circ h_3$ and $h_2\circ h_4$ must be compatible and so  is defined.

Now, writing *h* for , and letting $\ell \in \mathrm {dom}\left( h\right)$, we have 
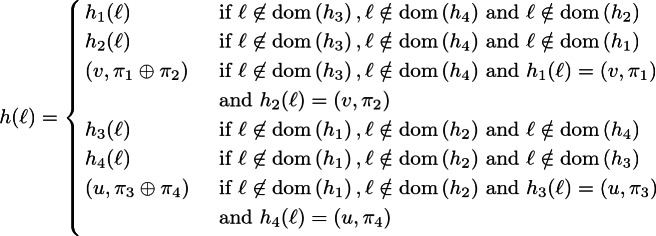
We can merge the first and fourth cases by noting that $h(\ell )=(h_1\circ h_3)(\ell )$ if $\ell \not \in \mathrm {dom}\left( h_2\circ h_4\right)$, and similarly for the second and fifth cases. We can also rewrite the last two cases by observing that $\ell \notin \mathrm {dom}\left( h_3\right)$ implies $h_1(\ell )=(h_1\circ h_3)(\ell )$, and so on, resulting in 
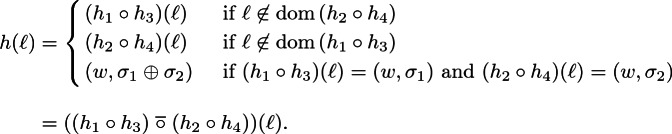
Now we show the main result. Suppose $s,h,\rho \models (A\circledast B)*(C\circledast D)$. This gives us , where $s,h_1,\rho \models A$ and $s,h_2,\rho \models B$ and $s,h_3,\rho \models C$ and $s,h_4,\rho \models D$. By Eq. (6), we have , which gives us exactly that $s,h,\rho \models (A*C)\circledast (B*D)$, as required.    $\square$

Next, we establish principles for distributing permissions over various connectives, in particular over the strong $*$, stated earlier as (3) in Sect. 2.

### Lemma 4.3

**(Permission distribution).** The following equivalences hold for all formulas *A* and *B*, and permissions $\pi$ and $\sigma$:

**Figure Figq:**
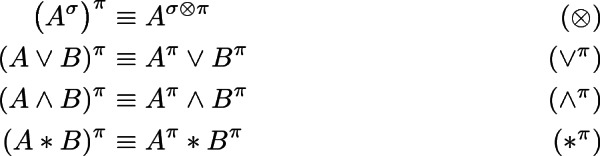

### Proof

We just show the most interesting case, ([Fig Figq]). First of all, we establish a corresponding model-theoretic property: for any permission $\pi$ and disjoint p-heaps $h_1$ and $h_2$, meaning $h_1 \circ h_2$ is defined,

7 $$\begin{aligned} \pi \cdot (h_1 \circ h_2) = (\pi \cdot h_1) \circ (\pi \cdot h_2). \end{aligned}$$

To see this, we first observe that for any $\ell \in \mathrm {dom}\left( h_1 \circ h_2\right)$, we have that either $\ell \in \mathrm {dom}\left( h_1\right)$ or $\ell \in \mathrm {dom}\left( h_2\right)$. We just show the case $\ell \in \mathrm {dom}\left( h_1\right)$, since the other is symmetric. Writing $h_1(\ell )=(v_1,\pi _1)$, and using the fact that $\ell \not \in \mathrm {dom}\left( h_2\right)$,

$$\begin{array}{c} \pi \cdot (h_1 \circ h_2)(\ell ) = (v_1,\pi \otimes \pi _1) = (\pi \cdot h_1)(\ell ) = ((\pi \cdot h_1) \circ (\pi \cdot h_2))(\ell ). \end{array}$$

Now for the main result, let *s*, *h* and $\rho$ be given. We have

$$\begin{array}[b]{c@{}l} &{} s,h,\rho \models (A*B)^\pi \\ \Leftrightarrow &{} h = \pi \cdot h'\text { and } s,h',\rho \models A * B \\ \Leftrightarrow &{} h = \pi \cdot h'\text { and } h' = h_1 \circ h_2 \text { and } s,h_1,\rho \models A \text { and } s,h_2,\rho \models B \\ \Leftrightarrow &{} h = \pi \cdot (h_1 \circ h_2) \text { and } s,h_1,\rho \models A \text { and } s,h_2,\rho \models B \\ \Leftrightarrow &{} h = (\pi \cdot h_1) \circ (\pi \cdot h_2) \text { and } s,h_1,\rho \models A \text { and } s,h_2,\rho \models B \qquad \qquad \text{ by } \text{(7) } \\ \Leftrightarrow &{} h = h_1' \circ h_2' \text{ and } s,h_1',\rho \models A^\pi \text{ and } s,h_2',\rho \models B^\pi \\ \Leftrightarrow &{} s,h,\rho \models A^\pi * B^\pi . \end{array}$$

   $$\square $$

We now establish the main principles for dividing and combining permissions formulas using $\circledast$. As foreshadowed in Sect. 2, the combination principle holds only for formulas that are conjoined with a nominal label (cf. Eq. (5)).

### Lemma 4.4

**(Permission division and combination).** For all formulas *A*, nominals $\alpha$, and permissions $\pi _1, \pi _2$ such that $\pi _1 \oplus \pi _2$ is defined:

**Figure Figr:**
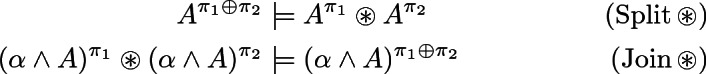

### Proof

**Case (**[Fig Figr]**):** Suppose that $s,h,\rho \models A^{\pi _1 \oplus \pi _2}$. We have $h = (\pi _1 \oplus \pi _2) \cdot h'$, where $s,h',\rho \models A$. That is, for any $\ell \in \mathrm {dom}\left( h\right)$, we have $h'(\ell ) = (v,\pi )$ say and, using the permissions algebra axiom (left-dist) from Definition 3.1,

$$ h(\ell ) = (v,(\pi _1\oplus \pi _2)\otimes \pi ) = (v,(\pi _1\otimes \pi )\oplus (\pi _2\otimes \pi )) . $$

Now we define p-heaps $h_1$ and $h_2$, both with domain exactly $\mathrm {dom}\left( h\right)$, by

$$ h_i(\ell ) = (v,\pi _i\otimes \pi ) \;\Leftrightarrow \; h'(\ell )=(v,\pi ) \qquad \text{ for } i \in \{1,2\}. $$

By construction, $h_1 = \pi _1 \cdot h'$ and $h_2 = \pi _2 \cdot h'$. Since $s,h',\rho \models A$, this gives us $s,h_1,\rho \models A^{\pi _1}$ and $s,h_2,\rho \models A^{\pi _2}$. Furthermore, also by construction, $h_1$ and $h_2$ are compatible, with . Thus $s,h,\rho \models A^{\pi _1} \circledast A^{\pi _2}$, as required.

**Case (**[Fig Figr]**):** First of all, we show that for any p-heap *h*,8To see this, we observe that for any $\ell \in \mathrm {dom}\left( h\right)$, writing $h(\ell )=(v,\pi )$ say,Now, for the main result, suppose $s,h,\rho \models (\alpha \wedge A)^{\pi _1} \circledast (\alpha \wedge A)^{\pi _2}$. We have  where $s,h_1,\rho \models (\alpha \wedge A)^{\pi _1}$ and $s,h_2,\rho \models (\alpha \wedge A)^{\pi _2}$. That is, , where $s,h_1',\rho \models \alpha \wedge A$ and $s,h_2',\rho \models \alpha \wedge A$. Thus $h_1'=h_2'=\rho (\alpha )$ and so, by (8), we have $h = (\pi _1 \oplus \pi _2)\cdot h_1'$, where $s,h_1',\rho \models \alpha \wedge A$. This gives us $s,h,\rho \models (\alpha \wedge A)^{\pi _1 \oplus \pi _2}$, as required.

Lastly, we state some useful principles for labels and the “jump” modality.

### Lemma 4.5

**(Labelling and jump).** For all formulas *A* and labels $\alpha$,

**Figure Figs:**
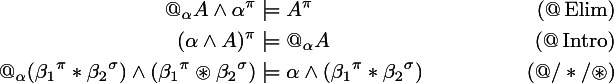

### Proof

We just show the case ([Fig Figs]), the others being easy. Suppose $s,h,\rho \models @_\alpha ({\beta _1}^\pi *{\beta _2}^\sigma ) \wedge ({\beta _1}^\pi \circledast {\beta _2}^\sigma )$, meaning that $s, \rho (\alpha ),\rho \models {\beta _1}^\pi *{\beta _2}^\sigma$ and $s,h, \rho \models {\beta _1}^\pi \circledast {\beta _2}^\sigma$. Then we have $\rho (\alpha )=(\pi \cdot \rho ({\beta _1}))\circ (\sigma \cdot \rho ({\beta _2}))$, while . Since $\circ$ is defined only when its arguments are disjoint p-heaps, we obtain that $h = \rho (\alpha ) = (\pi \cdot \rho ({\beta _1}))\circ (\sigma \cdot \rho ({\beta _2}))$. Thus $s,h,\rho \models \alpha \wedge ({\beta _1}^\pi *{\beta _2}^\sigma )$.   $\square$

## Concurrent Program Verification Examples

In this section, we demonstrate how $\mathsf {SL}_{\mathsf {LP}}$ can be used in conjunction with the usual principles of $\mathsf {CSL}$ to construct verification proofs of concurrent programs, taking three examples of increasing complexity.

**Fig. 2.**
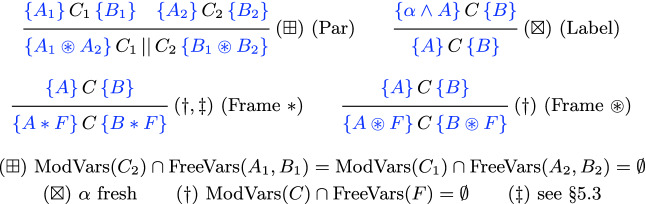
The key $\mathsf {CSL}$ proof rules used in our examples; not shown are standard rules for consequence, conditionals, load/store, etc. The fresh-labelling rule (Label) and combination of both weak (Frame $\circledast$) and strong (Frame $*$) frame rules are novel to our approach. We require weak conjunction $\circledast$ for the parallel rule (Par).

Our examples all operate on *binary trees* in memory, defined as usual in separation logic (again note the use of $*$ rather than $\circledast$): 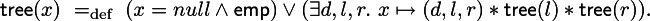
Our proofs employ (a subset of) the standard rules of $\mathsf {CSL}$—with the most important being the concurrency rule from the Introduction, the separation logic *frame rules* for both $*$ and $\circledast$, and a new rule enabling us to introduce fresh labels into the precondition of a triple (similar to the way Hoare logic usually handles existential quantifiers). These key rules are shown in Fig. 2. We simplify our Hoare triple to remove elements to handle function call/return and furthermore omit the presentation of the standard collection of rules for consequence, load, store, if-then-else, assignment, etc.; readers interested in such aspects can consult [1]. Both of our frame rules have the usual side condition on modified program variables. The strong frame rule (Frame $*$) has an additional side condition that will be discussed in Sect. 5.3; until then it is trivially satisfied.

### Parallel Read

**Fig. 3.**
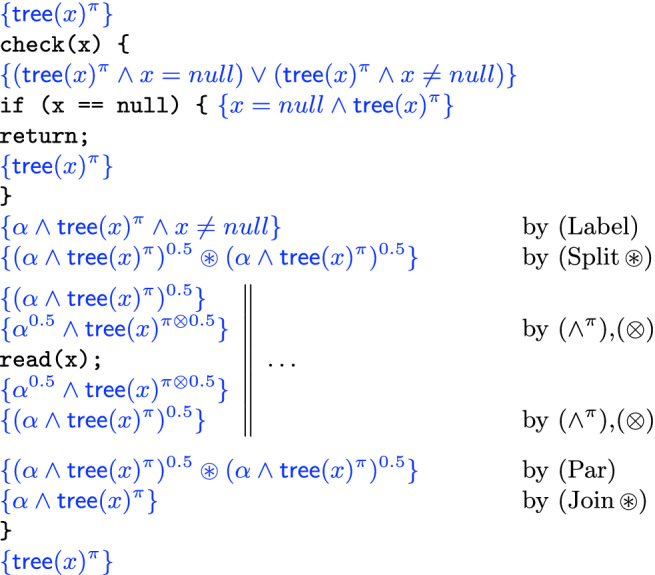
Verification proof of program $\texttt {check}$ in Example 5.1.

Consider the following program: 
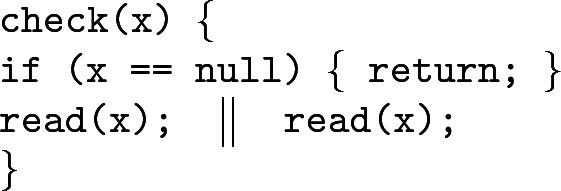


This is intended to be a straightforward example where we take a tree rooted at $\texttt {x}$ and, if $\texttt {x}$ is non-null, split into parallel threads that run the program $\texttt {read}$ on $\texttt {x}$, and whose specification is . We prove that $\texttt {check}$ satisfies the specification ; the verification proof is in Fig. 3. The proof makes use of the basic operations of our theory: labelling, splitting and joining. The example follows precisely these steps, starting by labelling the formula $\mathsf {tree}(x)^\pi \wedge x\ne null$ with $\alpha$. The concurrency rule (Par) allows us to put formulas back together after the parallel call, and the two copies $(\alpha \wedge \mathsf {tree}(x)^\pi )^{0.5}$ that were obtained are glued back together to yield $\mathsf {tree}(x)^\pi$, since they have the same label.

### Parallel Tree Processing (Le and Hobor [24])

**Fig. 4.**
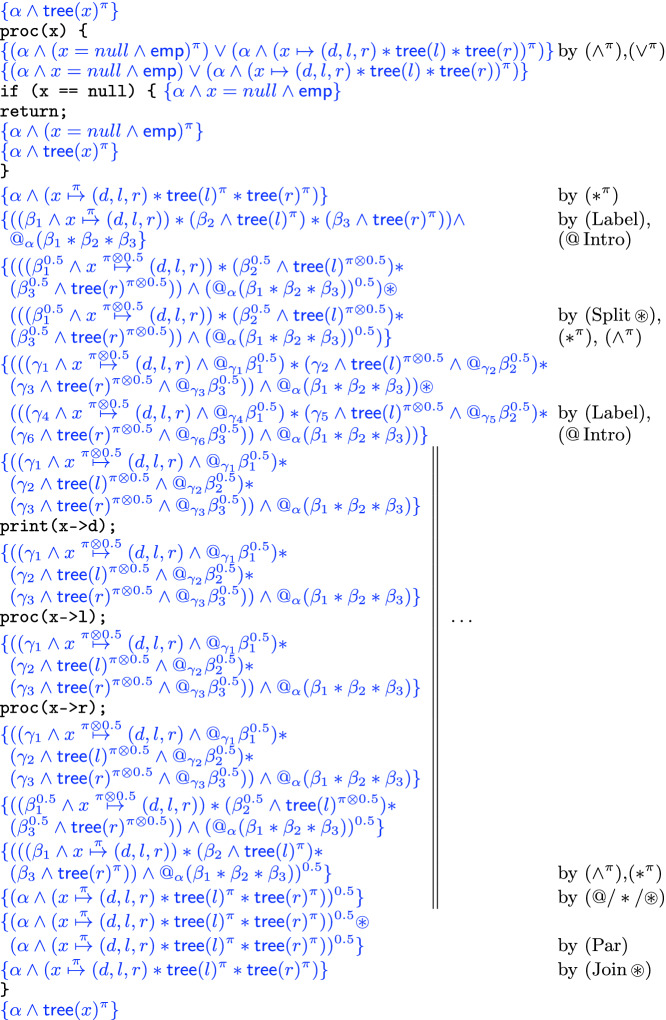
Verification proof of Le and Hobor’s program from [24] in Example 5.2.

Consider the following program, which was also employed as an example in [24]: 
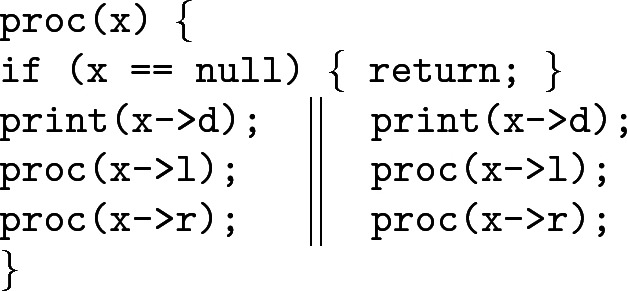


This code takes a tree rooted at $\texttt {x}$ and, if $\texttt {x}$ is non-null, splits into parallel threads that call $\texttt {proc}$ recursively on its left and right branches. We prove, in Fig. 4, that $\texttt {proc}$ satisfies the specification . First we unroll the definition of $\mathsf {tree}(x)$ and distribute the permission over Boolean connectives and $*$. If the tree is empty the process stops. Otherwise, we label each component with a new label and introduce the “jump” statement $@_\alpha (\beta _1*\beta _2*\beta _3)$, recording the decomposition of the tree into its three components. Since such statements are *pure*, i.e. independent of the heap, we can “carry” this formula along our computation without interfering with the frame rule(s). Now that every subregion is labelled, we split the formula into two copies, each with half share, but after distributing ${0.5}$ over $*$ and $\wedge$ we end up with half shares in the labels as well. We relabel each subregion with new “whole” labels, and again introduce pure @-formulas that record the relation between the old and the new labels. At this moment we enter the parallel threads and recursively apply $\texttt {proc}$ to the left and right subtrees of $\texttt {x}$. Assuming the specification of $\texttt {proc}$ for subtrees of $\texttt {x}$, we then retrieve the original label $\alpha$ from the trail of crumbs left by the @-formulas. We can then recombine the $\alpha$-labelled threads using ([Fig Figr]) to arrive at the desired postcondition.

### Cross-thread Data Transfer

Our previous examples involve only “isolated tank” concurrency: a program has some resources and splits them into parallel threads that do not communicate with each other before—remembering Humpty Dumpty!—ultimately re-merging. For our last example, we will show that our technique is expressive enough to handle more sophisticated kinds of sharing, in particular inter-thread coarse-grained communication. We will show that we can not only share read-only data, but in fact prove that one thread has acquired the full ownership of a structure, even when the associated root pointers are not easily exposed.

To do so, we add some communication primitives to our language, together with their associated Hoare rules. Coarse-grained concurrency such as locks, channels, and barriers have been well-investigated in various flavours of concurrent separation logic [19, 26, 31]. We will use a channel for our example in this section but with simplified rules: the Hoare rule for a channel *c* to send message number *i* whose message invariant is $R^c_i$ is  $\texttt {send(}c,x\texttt {)}$ , while the corresponding rule to receive is   . We ignore details such as identifying which party is allowed to send/receive at a given time [14] or the resource ownership of the channel itself [18].

These rules interact poorly with the strong frame rule from Fig. 2: 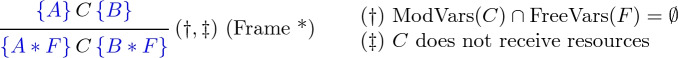
The revealed side condition $(\ddag )$ means that *C* does not contain any subcommands that “transfer in” resources, such as unlock, receive, etc.; this side condition is a bit stronger than necessary but has a simple definition and can be checked syntactically. Without $(\ddag )$, we can reach a contradiction. Assume that the current message invariant $R^{\texttt {c}}_i$ is , which has been sent by thread B. Now thread A, which had the other half of , can reason as follows: 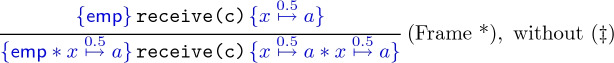
The postcondition is a contradiction as no location strongly separates from itself. However, given $(\ddag )$ the strong frame rule can be proven by induction.

The consequence of $(\ddag )$, from a verification point of view, is that when resources are transferred in they arrive *weakly separated*, by $\circledast$, since we must use the weak frame rule around the receiving command. The troublesome issue is that this newly “arriving” state can thus $\circledast$-overlap awkwardly with the existing state. Fortunately, judicious use of labels can sort things out.

**Fig. 5.**
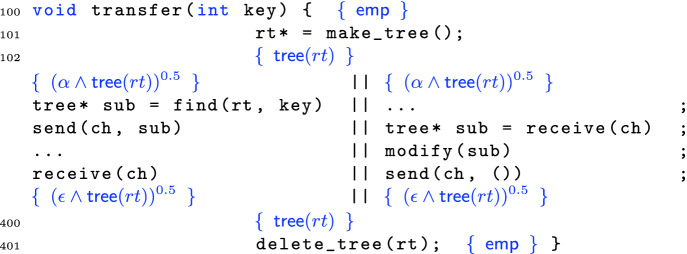
Verification proof of the top and bottom of transfer in Example 5.3.

**Fig. 6.**
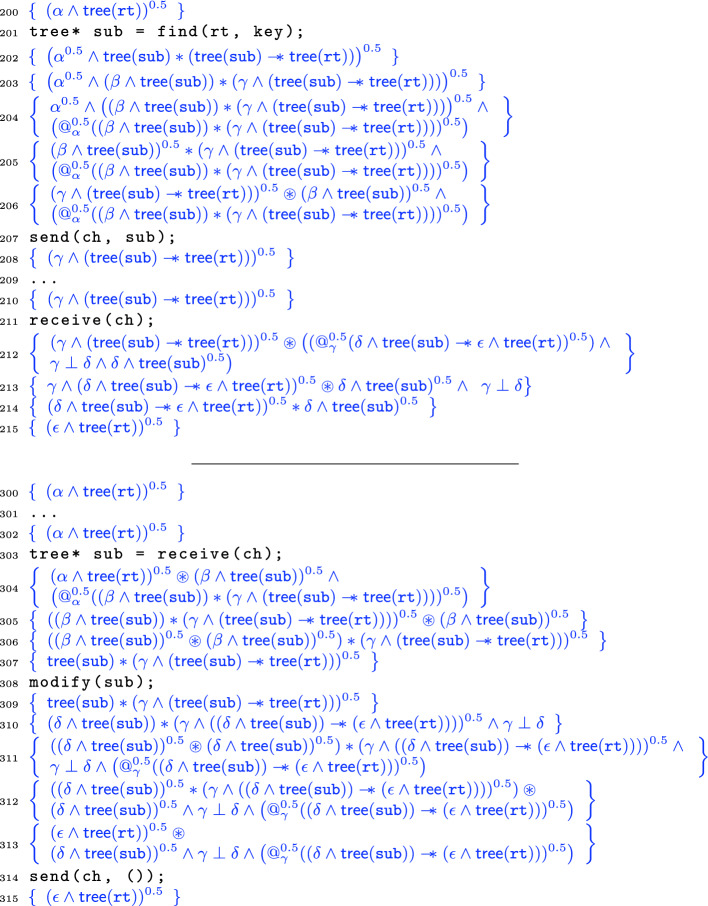
Verifications of the left (top) and right (bottom) threads of transfer.

Consider the code in Fig. 5. The basic idea is simple: we create some data at the top (line 101) and then split its ownership 50-50 to two threads. The left thread finds a subtree, and passes its half of that subtree to the right via a channel. The right thread receives the root of that subtree, and thus has full ownership of that subtree along with half-ownership of the rest of the tree. Accordingly, the right thread can modify that subtree before notifying the left subtree and passing half of the modified subtree back. After merging, full ownership of the entire tree is restored and so on line 401 the program can delete it. Figure 5 only contains the proof and line numbers for the top and bottom shared portions. The left and the right thread’s proofs appear in Fig. 6.

By this point the top and bottom portions of the verification are straightforward. After creating the tree  at line 102, we introduce the label $\alpha$, split the formula using ([Fig Figr]), and then pass  to both threads. After the parallel execution, due to the call to modify(sub) in the right thread, the tree has changed in memory. Accordingly, the label for the tree must also change as indicated by the  in both threads after parallel processing. These are then recombined on line 400 using the re-combination principle ([Fig Figr]), before the tree is deallocated via standard sequential techniques.

Let us now examine the more interesting proofs of the individual threads in Fig. 6. Line 201 calls the find function, which searches a binary tree for a subtree rooted with key key. Following Cao *et al.* [13] we specify find as follows: 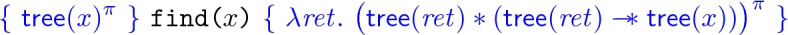
Here  is bound to the return value of find, and the postcondition can be considered to represent the returned subtree  separately from the tree-with-a-hole , using a  style to represent replacement as per Hobor and Villard [20]. This is the invariant on line 202.

Line 203 then attaches the fresh labels $\beta$ and $\gamma$ to the $*$-separated subparts, and line 204 snapshots the formula current at label $\alpha$ using the @ operator;  should be read as “when one has a $\pi$-fraction of $\alpha$, *P* holds”; it is definable using @ and an existential quantifier over labels. On line 205 we forget (in the left thread) the label $\alpha$ for the current heap for housekeeping purposes, and then on line 206 we weaken the strong separating conjunction $*$ to the weak one $\circledast$ before sending the root of the subtree sub on line 207.

In the transfer program, the invariant for the first channel message is 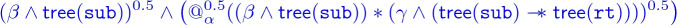
In other words, half of the ownership of the tree rooted at sub plus the (pure) @-fact about the shape of the heap labeled by $\alpha$. Comparing lines 206 and 208 we can see that this information has been shipped over the wire (the @-information has been dropped since no longer needed). The left thread then continues to process until synchronizing again with the receive in line 211.

Before we consider the second synchronization, however, let us instead jump to the corresponding receive in the right thread at line 303. After the receive, the invariant on line 304 has the (weakly separated) resources sent from the left thread on line 206. We then “jump” label $\alpha$ using the @-information to reach line 305. We can redistribute the $\beta$ inside the $*$ on line 306 since we already know that $\beta$ and $\gamma$ are disjoint. On line 307 we reach the payoff by combining both halves of the subtree sub, enabling the modification of the subtree in line 308.

On line 310 we label the two subheaps, and specialize the magic wand so that given the specific heap $\delta$ it will yield the specific heap $\epsilon$; we also record the pure fact that $\gamma$ and $\delta$ are disjoint, written . On line 311 we snapshot $\gamma$ and split the tree sub 50-50; then on line 312 we push half of sub out of the strong $*$. On line 313 we combine the subtree and the tree-with-hole to reach the final tree $\epsilon$. We then send on line 314 with the channel’s second resource invariant: 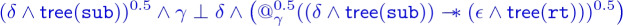
After the send, on line 315 we have reached the final fractional tree $\epsilon$.

Back in the left-hand thread, the second send is received in line 211, leading to the weakly-separated postcondition in line 212. In line 213 we “jump” label $\gamma$, and then in line 214 we use the known disjointness of $\gamma$ and $\delta$ to change the $\circledast$ to $*$. Finally in line 215 we apply the magic wand to reach the postcondition.

## Conclusions and Future Work

We propose an extension of separation logic with fractional permissions [4] in order to reason about sharing over arbitrary regions of memory. We identify two fundamental logical principles that fail when the “weak” separating conjunction $\circledast$ is used in place of the usual “strong” $*$, the first being distribution of permissions—$A^\pi \circledast B^\pi \not \models (A \circledast B)^\pi$—and the second being the re-combination of permission-divided formulas, $A^\pi \circledast A^\sigma \not \models A^{\pi \oplus \sigma }$. We avoid the former difficulty by *retaining* the strong $*$ in the formalism alongside $\circledast$, and the latter by using nominal *labels*, from hybrid logic, to record exact aliasing between read-only copies of a formula.

The main previous work addressing these issues, by Le and Hobor [24], uses a combination of permissions based on *tree shares* [17] and semantic side conditions on formulas to overcome the aforementioned problems. The *rely-guarantee* separation logic in [30] similarly restricts concurrent reasoning to structures described by precise formulas only. In contrast, our logic is a little more complex, but we can use permissions of any kind, and do not require side conditions. In addition, our use of labelling enables us to handle examples involving the transfer of data structures between concurrent threads.

On the other hand, we think it probable that the kind of examples we consider in this paper could also be proven by hand in at least some of the verification formalisms derived from $\mathsf {CSL}$ (e.g. [16, 22, 27]). For example, using the “concurrent abstract predicates” in [16], one can explicitly declare shared regions of memory in a fairly ad-hoc way. However, such program logics are typically very complicated and, we believe, quite unlikely to be amenable to automation.

We feel that the main appeal of the present work lies in its relative simplicity—we build on standard $\mathsf {CSL}$ with permissions and invoke only a modest amount of extra syntax—which bodes well for its potential automation (at least for simpler examples). In practical terms, an obvious way to proceed would be to develop a prototype verifier for concurrent programs based on our logic $\mathsf {SL}_{\mathsf {LP}}$. An important challenge in this area is to develop heuristics—e.g., for splitting, labelling and combining formulas—that work acceptably well in practice.

An even greater challenge is to move from *verifying* user-provided specifications to *inferring* them automatically, as is done e.g. by Facebook Infer. In separation logic, this crucially depends on solving the *biabduction* problem, which aims to discover “best fit” solutions for applications of the frame rule [9, 11]. In the $\mathsf {CSL}$ setting, a further problem seems to lie in deciding how applications of the concurrency rule should divide resources between threads.

Finally, automating the verification approach set out in this paper will likely necessitate restricting our full logic to some suitably tractable fragment, e.g. one analogous to the well-known *symbolic heaps* in standard separation logic (cf. [2, 15]). The identification of such tractable fragments is another important theoretical problem in this area. It is our hope that this paper will serve to stimulate interest in the automation of concurrent separation logic in particular, and permission-sensitive reasoning in general.
